# Physical activity-related health competence and symptom burden for exercise prescription in patients with multiple myeloma: a latent profile analysis

**DOI:** 10.1007/s00277-023-05326-y

**Published:** 2023-06-24

**Authors:** Rea Kuehl, Maximilian Koeppel, Hartmut Goldschmidt, Imad Maatouk, Friederike Rosenberger, Joachim Wiskemann

**Affiliations:** 1 Working Group Exercise Oncology, Division of Medical Oncology, National Center for Tumor Diseases (NCT) Heidelberg, Im Neuenheimer Feld 460, 69120 Heidelberg, Germany; 2 Department of Internal Medicine V, University Hospital Heidelberg and National Center for Tumor Diseases (NCT) Heidelberg, Heidelberg, Germany; 3 Department of General Internal Medicine and Psychosomatics, University Hospital Heidelberg, Heidelberg, Germany; 4 Section of Psychosomatic Medicine, Psychotherapy and Psychooncology, Department of Internal Medicine II, Julius-Maximilian University Wuerzburg, Wuerzburg, Germany; 5 Division of Health Sciences, German University of Applied Sciences for Prevention and Health Management, Saarbruecken, Germany

**Keywords:** Exercise, Physical activity, Cancer, Multiple myeloma, Symptom burden, PAHCO

## Abstract

**Abstract:**

The purpose of this study is to ensure best possible supply of exercise therapy to patients with multiple myeloma (MM); it is helpful to identify patient groups with similar symptom burden and physical activity–related health competences (PAHCO). Latent profile analyses (LPA) of MM patients were used to identify profiles of patients with similar PAHCO and symptom burden. Analysis of variance was applied to investigate group differences in important covariates. *N* = 98 MM patients (57% male, age 64 ± 9 years) could be assigned to three distinct PAHCO profiles: 46% were patients with high PAHCO, 48% patients with moderate, and 5% were patients with low PAHCO. The mean probability to be assigned to a certain profile was over 99%. The first group showed significant higher physical activity (PA) and lower comorbidities. Regarding symptom burden, three different profiles exist, including group one (32% of patients) with very low symptom burden, profile two (40%) with medium symptom burden, and group three (15%) with very high symptom burden (mean probability ≥ 98%). Patients in profile one had a lower number of treatment lines compared to the other profiles. Patients who were assigned to the high PAHCO profile were more likely to display a milder symptoms profile. In this exploratory analysis, we identified different patient profiles for PAHCO and symptom burden that may be used to individualize exercise recommendations and supervision modalities in MM patients. PAHCO and symptom burden level may be used to stratify MM patients in order to provide more personalized and effective exercise counseling. The profiles require individualized exercise recommendations and different supervision modalities, including educational instructions tailored particularly to every patient’s needs, according to their PAHCO and symptom profile.

**Trial registration number:**

NCT04328038.

**Supplementary Information:**

The online version contains supplementary material available at 10.1007/s00277-023-05326-y.

## Introduction

Physical exercise interventions in cancer patients have shown to be safe, feasible, and an effective option to counteract a wide range of negative cancer- or therapy-related side effects, such as cancer-related fatigue (CRF), loss of physical function, peripheral neuropathy, and subsequently, reduced quality of life (QOL). A large body of evidence comprising hundreds of RCTs demonstrates these positive effects [1]. This evidence has resulted in the development of international guidelines in recent years. For example, the American College of Sports Medicine (ACSM) recommend 90–150 min moderate physical activity (PA) and two weekly sessions of resistance exercise [2]. However, exercise programs should always be adapted to the individual patient and its personal profile of side effects, previous experience with exercise, and physical abilities [3]. This is particularly true for patients under or after intensive treatment, e.g., stem cell transplantation, or long lasting treatment, as it is typically in multiple myeloma (MM) patients.

Despite large improvements of therapeutic options and subsequently improvements in survival rates, MM remains an incurable disease in most patients, requiring long lasting therapies [4–6]. Besides high symptom burden due to the cumulative toxicities of therapies [7, 8], the high prevalence of osteolytic bone disease with high fracture risk [9, 10] poses major challenges when providing PA or exercise advice. The physical functioning in newly diagnosed MM patients is already lower than in healthy populations [11]. Nevertheless, first randomized exercise intervention studies have been conducted with MM patients, who demonstrated positive effects on different outcomes, e.g., QOL and CRF [12–15] and a higher PA level was associated with lower CRF, fewer side effects, and better QOL [16]. However, the effects seemed to be less clear as in other entities.

Patients with MM seem highly motivated to be physically active. Recent studies showed that 75% of MM patients would like to increase their exercise level, 59% would like to receive advice regarding PA [17], and 55% were interested in exercise programs [18]. Almost all (97%) clinical hematologists treating MM patients agreed that PA is important for MM patients with benefits for, e.g., QOL and CRF [19]. Despite their high motivation, only 12–25% of MM patients are physically active [17, 18] and the activity level declines during the course of the therapy [18, 20]. This can be attributed to individual barriers, such as high levels of CRF, pain, uncertainties concerning the fracture risk, weakness and neuropathy could be identified [17, 20]. Seventy-one percent of MM patients reported that a combination rather than a single barrier prevented them from PA [17].

From a health psychological perspective, patients need certain skills, knowledge, and motivation in order to engage in regular exercise or PA. To explain this, the model of PA-related health competences (PAHCO) was developed [21–23]. It is based on health literacy models [24] and is grounded on a pragmatic understanding of competence and contains the three sub-competences: movement competence (movement-related requirements of PA, e.g., motor skills and abilities), control competence (CC, describes the degree a person is capable of utilizing their knowledge regarding exercise, e.g., control physical load), and PA-specific self-regulation competence (SC, motivational and volitional abilities, e.g., self-control) [23, 25]. It could be shown that the individual competence was linked to the regular amount of leisure time PA in a mixed sample of cancer patients [26]. Furthermore, the CC was directly related to physical fitness [25].

Although, MM patients are highly motivated to be physically active, high symptom burden and low PAHCO competences seem to prevent them from being physically active. Furthermore, the complex situation with high symptom burden presents a great challenge for exercise therapists to provide exercise advice and choose the level of support patients need to initiate and maintain an exercise program. Therefore, the purpose of this study was to identify patient groups (profiles) with (a) similarity in PAHCO and (b) similar symptom profiles, such as CRF, pain, and low physical functioning. This approach could result in a more personalized exercise counseling, including selection of appropriate exercise content matching the individual symptom profile and further exercise support, e.g., supervision or including motivational aspects.

## Methods

### Participants

This is a cross-sectional study conducted at the National Center for Tumor Diseases (NCT) Heidelberg, Germany, between June 2020 and October 2020. The study protocol was approved by the ethics committee of the University Hospital Heidelberg (S-875/2019) and is registered at ClinicalTrials.gov (NCT04328038). Patients were eligible to participate when they met the following inclusion criteria: MM or a precursor, e.g., smoldering myeloma (SMM), ≥ 18 years, mobile enough to conduct exercise/ECOG PS ≤ 2, and able to follow the study instructions. The participants were recruited during clinical outpatient visits or during ambulatory treatment. Patients were informed about the study from their treating hematologist and signed a written informed consent form before completing the survey. They completed the paper-and-pencil survey in the waiting rooms of the NCT or during treatment at the day care unit.

### Measures

Sociodemographic and medical information was partly obtained from medical records and partly self-reported in the survey. Sociodemographic information contained age, sex, education, family status, and employment. Medical data contained diagnosis, time since diagnosis, diagnosis of bone disease, therapy, treatment line, and number of comorbidities.

#### EORTC QOL-C30

QOL was assessed with the 30-item questionnaire of the European Organization for Research and Treatment of Cancer (EORTC QOL-C30), version 3.0 [27]. Besides a global health and QOL scale, the EORTC QLQ-C30 consists of five functional scales (physical, role, cognitive, emotional, and social functioning) which were included in the further analysis. The three multi-item symptom scales (fatigue, nausea, and vomiting) and six single item scales (dyspnea, appetite loss, sleep disturbance, constipation, diarrhea, and the financial impact of the illness) were not included in the further analysis. We excluded the multi-item symptom scale for fatigue since we applied the EORTC QLQ FA12 to assess CRF. The scales for nausea and vomiting were also excluded because they were not effectively addressed by exercise therapy. The single-item scales were excluded due to a lack of validity. All items were answered following a four-stage Likert scale (from “not at all” to “very much”). All scores were derived according to the EORTC scoring manual and were transformed to range from 0 to 100; thus, high scores equal high QOL, high functioning, and high symptoms. The questionnaire was validated in samples with myeloma disease [28].

#### EORTC QOL FA12

The EORTC FA12 is a multidimensional self-reporting screening tool to assess CRF. The tool was developed by the EORTC quality of life group and is used in conjunction with the EORTC QLQ-C30 [29]. The questionnaire divides CRF into 3 subscales using a total of 12 items: physical CRF (PF, 5 items), emotional CRF (EF, 3 items), and cognitive CRF (CF, 2 items). The remaining 2 items serve as global indicators for impairment in performing daily life activities as well as the social sequelae of CRF, but they do not belong to a single subscale. All items were answered according to a four-stage Likert scale (from “not at all” to “very much”). Reported Cronbach’s alphas were good for all three dimensions with 0.88 to 0.90 for PF, 0.87 to 0.88 for EF, and 0.79 to 0.82 for CF [29].

#### Brief pain inventory (BPI)

Pain was assessed with the brief pain inventory (BPI). It measures both pain severity (4 items) and pain interference on functioning (7 items) using a Likert scale (from 0 to 10) [30]. The BPI is a valid and reliable tool for pain measurement in cancer patients with bone metastases [31, 32].

#### Distress

Distress (distress, anxiety, depression, anger) were assessed with the Distress-Thermometer (VAS 0–10), developed by the National Comprehensive Cancer Network (NCCN) as a screening tool [33, 34].

#### Physical activity–related health competence (PAHCO)

The questionnaire is based on the PAHCO model outlined above and assesses specific facets of the PAHCO, specifically addressing an individual’s aptitude to effectively utilize PA in order to optimize their overall health. The questionnaire consists of 13 items that comprised 3 latent factors: PA-specific mood regulation (MR, 4 items), CC for physical training (6 items) and PA-specific SC (3 items). [25] In contrast to the model, the PAHCO Questionnaire has no items to assess movement competence, but instead it focuses on the implementation (SC) and utilization (MR) of health enhancing PA, the control of physical load via body signals, as well as knowledge about the effects (CC). All items were answered on a four-stage Likert scale with possible responses ranging from “disagree completely” (1) to “agree completely” (4). McDonald’s omega was good for all three dimensions with 0.94 for MR, 0.93 for CC, and 0.92 for SC in cancer patients [26]. The original PAHCO questionnaire was developed and validated on two German samples, one sample includes oncological patients during rehabilitation period [25].

#### Short questionnaire to assess health-enhancing physical activity (SQUASH)

The SQUASH is a commonly used instrument to assess PA behavior in adults, comparing the PA levels of individuals and evaluating compliance with PA guidelines [35]. The SQUASH was developed by the Dutch National Institute of Public Health and the Environment [36]. It relies on self-reports, assessing 4 main domains: (a) commuting activities, (b) leisure time activities, (c) household activities, and (d) activities at work and school, which are evaluated based on an average week. The participants rate the amount of time they spent on each domain using 3 main queries: days per week, average time per day, and intensity (effort). To quantify the intensity of the activity, a metabolic equivalent task (MET) value, based on Ainsworth’s compendium of PA [37] is assigned to the activities; subsequently, depending on the effort reported, the activities receive an intensity score and total score. For the forthcoming analysis only the information about leisure time activities was utilized since this is the PA dimension with the most degrees of freedom and constitutes a patient’s voluntary choice.

### Statistical methods

In order to identify distinctive profiles of patients, we conducted 2 separate latent profile analyses (LPA). In the first LPA the model was fitted to the 3 factors of the PAHCO-questionnaire to identify groups of patients with distinct PAHCO. In the second LPA we fitted the LPA to the EORTC QLQ-30, -FA-12, distress, and BPI scores to identify groups of patients that differ in the composition of their symptoms and symptom burden. While results of the first LPA were used to identify the diverging needs in patients on how the exercise therapy should be applied and supervised, the results of the second LPA are important to tailor the specific exercise program. We did not have any prior hypothesis regarding the structure of the profiles; thus, this is solely an exploratory approach.

LPA is a probabilistic procedure that is closely related to cluster analysis. In contrast to cluster analysis, however, an individual is not exclusively assigned to one class. Instead probabilities are calculated that indicate how well the characteristics of an individual fit the various classes. Eventually, the individual is assigned to the profile with the highest probability. Information criteria were used to decide the optimal number of classes by applying the procedure from Akogul and Erisoglu [38]. In addition to this analytic approach, the validity of the resulting profiles was checked in terms of how reasonable the composition of characteristics of these profiles is. Profiles that contain less than 5% of the sample are considered spurious and may indicate the extraction of too many profiles [39]. The LPAs were conducted in the statistical program R using the packages Mclust [40] and tidyLPA [41].

After fitting the models and examining the distinct profiles, we applied ANOVA (for continuous variables) and Fishers exact test (for categorical variables) to investigate to what extend the profiles would differ in their levels of PA, age, gender, number of treatment line, number of comorbidities, and stability of the bones. Due to the exploratory character of the study, no correction for multiple testing was done. Consequently, the results of the ANOVA have to be interpreted with caution, and low *p*-values only show potential tendencies in the data but must not be mistaken as confirming a hypothesis. The intention behind the ANOVA was to examine if the data and the clusters are logical.

In case of the EORTC Questionnaires, missing data were handled according to the EORTC manual [40]. For the other questionnaires we applied a multiple imputation procedure based on the maximum-expectation method. Since the proportion of missing data did not exceed 3% for any variable, the impact of the imputed values have on the inferences is expected to be negligible [41].

## Results

### Patients

From *n* = 170 screened patients, *n* = 10 did not fulfill inclusion criteria and *n* = 20 were not interested in the study. From *n* = 140 questionnaires we handed out, *n* = 132 (94%) were returned. After excluding *n* = 6 patients who did not answer one of the questionnaires, the final sample consisted of *n* = 126 patients (90%) with MM/SMM. Twenty-eight of these (22%) were diagnosed with SMM and excluded for this analysis since SMM is considered a preliminary form of MM that does not necessarily demand medical treatment but is under surveillance. Thus, the final sample consisted of *n* = 98 patients with MM (see Online Resource 1 for Consort diagram). The patient characteristics are provided in Table 1. The mean age of the patients was 64.0 (SD = 9.2, range 38–84) with *n* = 42 of the patients (43%) being female. Sixty-seven patients (68%) have been diagnosed with bone disease and *n* = 8 (8%) already underwent surgery because of their bone disease. In *n* = 7 patients (7%) the bony lesions were considered unstable in terms of fracture risk. Sixty-six patients (67%) underwent autologic hematopoietic stem cell transplantation. Seventy-three patients underwent chemotherapy (75%), *n* = 35 patients radiotherapy (36%), *n* = 47 patients immunotherapy (48%), and *n* = 10 patients did not receive any form of treatment (10%, newly diagnosed MM patients). Thirty patients (31%) were employed.

**Table 1:** Sociodemographic and medical data (*n* = 98)

	*n*	%	
Gender			
Male	56	57.1%	
Female	42	42.9%	
Age in years (mean)	64.0	SD: 9.2	Range: 38–84
Family status			
Single	5	5.1%	
Married/partnership	80	81.6%	
Divorced/separated	8	8.2%	
Widowed	4	4.1%	
Missing	1	1.0%	
Education			
No degree	1	1.0%	
Secondary school	26	26.5%	
Middle school	20	20.4%	
High school	12	12.2%	
University degree	39	39.8%	
Employment			
Yes	30	30.6%	
No	58	59.2%	
Missing	10	10.2%	
Time since diagnosis (years)	3.9 (median)		Range: 0.07–18,0
	4.3 (mean)		
Bone disease			
Yes	67	68.4%	
No Missing	30 1	30.6% 1.0%	
Therapy			
No	10	10.2%	
Chemotherapy	73	74.5%	
Radiation	35	35.7%	
Immunotherapy	47	48.0%	
Autologous stem cell transplantation	66	67.3%	
More than one autologous stem cell transplantation	31	31.6%	
Treatment line			
No	9	9.2%	
1	64	65.3%	
2	11	11.2%	
3	7	7.1%	
≥ 3	7	7.1%	
Number comorbidities			
No	17	17.3%	
1	34	34.7%	
2	27	27.6%	
3	19	19.4%	
4	1	1.0%	

### Latent profile analysis

Latent profile models were fit to the data. Fit indices for each model and model comparison statistics of both analyses are presented in the Online Resource 2. For the PAHCO variables, a four profile solution displayed optimal fit according to the AHP procedure. However, inspecting the competing models, one of the four profiles showed considerable overlap with another profile. Therefore, we chose the three profile solution which provided three substantially distinct profiles (P-profiles).

### PAHCO

**Fig. 1:**
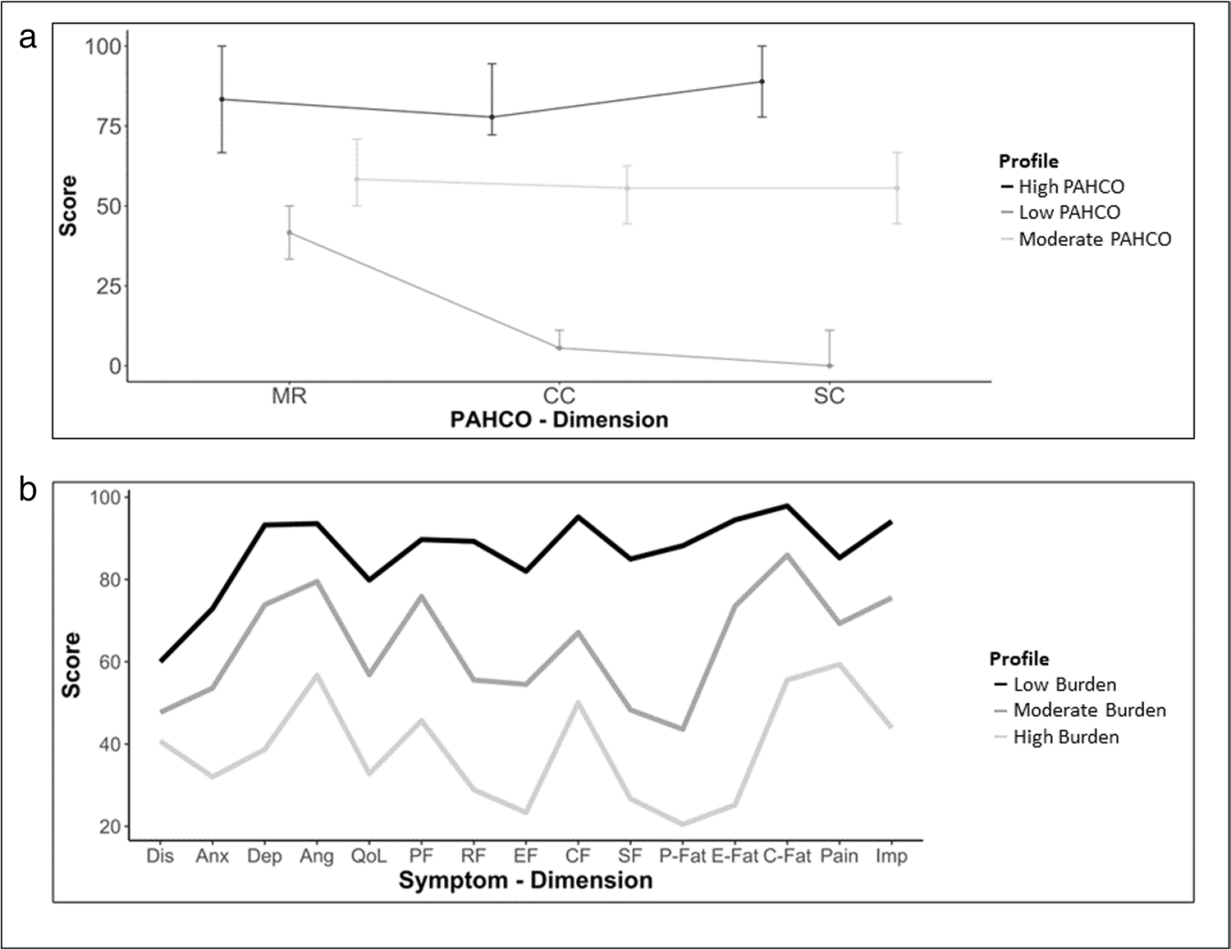
Means of latent profiles for **a** PAHCO and **b** symptoms. PAHCO, physical activity-related health competences; MR, mood regulation; CC, control competence; SC, self-regulation competence; Dis, distress; Anx, anxiety; Dep, depression; Ang, anger; QoL, quality of life global score; PF, physical function; RF, role function; EF, emotional function; CF, cognitive function; SF, social function; P-Fat, physical fatigue; E-Fat, emotional fatigue; C-Fat, cognitive fatigue; Pain, pain level (BPI); Imp, impairment through pain (BPI)

P-profile one is composed of 46% of the sample (*n* = 45) and represents patients with high PAHCO. P-profile two is composed of 48% of the sample (*n* = 47) and represents patients with moderate PAHCO; and P-profile three consists of 5% (*n* = 5) of the patient cohort, having low PAHCO. The overall means and the conditional profile means of the three-profile model are displayed in Table 2 and Fig. 1a. As portrayed in Fig. 1a, all profiles show high coherence regarding the PAHCO dimensions. Patients showed a high median probability to be assigned to a certain profile with 99% for the first and second groups and 100% for the third group (mean: 91%, 95%, 98%). P-profile one and three display no overlap in the assigning probabilities (< 0.1%) (see Online Resource 3). Due to the high distinction and the logical validity of the last group, we opted to keep the group despite the rather small proportion of patients assigned to it.

**Table 2:** Overall sample median (interquartile range) and profile conditional response means (standard deviation) for PAHCO-dimensions

	*n* (%)	MR	CC	SC
Sample	97 (99)	66.7 (58.3, 91.7)	66.7 (50.0, 77.8)	66.7 (55.6, 88.9)
*3-Profile Solution*				
Profile 1	45 (46)	83.3 (66.7, 100)	77.8 (72.2, 94.4)	88.9 (77.8, 100)
Profile 2	47 (48)	58.3 (50.0, 70.8)	55.6 (44.4, 62.6)	55.6 (44.4, 66.7)
Profile 3	5 (5)	41.7 (33.3, 50.0)	5.6 (5.6, 11.1)	0 (0, 11.1)

*PAHCO*, physical activity-related health competences; *n*, sample size; *MR*, mood regulation; *CC*, control competence; *SC*, self-regulation competence

### Symptom burden

In the case of the symptom scales, the AHP-procedure denominated the three-profile solution as optimal (S-profiles). Since 13 patients (13%) were missing complete data from the distress thermometer, the classes were only estimated for 87% (*n* = 85) of the patients. S-profile one includes 40% (*n* = 39) of the sample and comprised patients with low symptom burden. S-profile two incorporates 32% (*n* = 31) of enrolled patients who display medium symptom burden and S-profile three includes 15% (*n* = 15) of patients with very high symptom burden. The overall means and conditional class means of the three-profile model are displayed in Table 3 and Fig. 1b. In Fig. 1b we flipped the items of the distress thermometer, the BPI, and EORTC-FA12 in order to improve readability so that higher value equals better health. All profiles show high mean assignment probabilities ranging from 98 to 100% (see Online Resource 3). The 3 profiles are distinct as it can be seen in Fig. 1b (due to the large number of dimensions, only the line plot is displayed here; for a plot including error bars showing standard deviations, please refer to Online Resource 5).

**Table 3:** Overall sample median (IQR) and class conditional response medians (IQR) for symptom dimensions

	*n* (%)	Dis	Anx	Dep	Ang	QoL	PF	RF	EF	CF	SF	P-Fat	E-Fat	C-Fat	Pain	Imp
Sample	98 (78)	5 (3,7)	4 (1.8, 7)	1 (0, 5)	1 (0,3)	66.7 (50,83.3)	80.0 (60, 93.3)	66.7 (33.3, 100)	58.3 (41.7, 83.3)	83.3 (54.2, 100)	66.7 (33.3, 95.8)	46.7 (13.3, 66.7)	22.2 (0, 44.4)	0 (0, 16.7)	2.6 (0.8, 4.4)	1.7 (0.3, 3.9)
*3-Profile solution*																
Profile 1	31 (32)	3 (3, 5.5)	2 (0, 5)	0 (0, 1)	0 (0,1)	83.3 (75.0, 84.4)	93.3 (80, 100)	100 (83.3, 100)	83.3 (70.83, 100)	100 (91.7, 100)	100 (66.7, 100)	13.3 (0, 20)	0 (0, 0)	0 (0, 0)	0.75 (0, 2.6)	0.1 (0, 1)
Profile 2	39 (40)	5 (4, 7)	5 (3, 7)	2 (0.5, 4.5)	1 (0,3)	58.3 (50, 66.7)	80.0 (66.7, 89.2)	66.7 (33.3, 66.7)	50.0 (41.7, 66.7)	66.7 (50.0, 83.3)	50 (33.3, 66.7)	60 (46.7, 66.7)	22.2 (11.1, 33.3)	16.7 (0, 16.7)	2.8 (1.5, 4.8)	2.1 (0.9, 3.4)
Profile 3	15 (15)	6 (4, 8)	8 (5.5, 8)	7 (5.5, 8)	5 (3,6)	33.3 (16.7, 45.8)	40.0 (26.7, 60.0)	16.7 (8.3, 33.3)	25 (12.5, 25)	50 (33.3, 66.7)	33.3 (8.3, 33.3)	80 (66.7 93.3)	66.7 (61.1, 94.4)	50 (16.7, 66.7)	4 (3, 5.4)	5.6 (4.0, 7.4)

*n*, sample size; *Dis*, distress; *Anx*, anxiety; *Dep*, depression; *Ang*, anger; *QoL*, quality of life global score; *PF*, physical function; *RF*, role function; *EF*, emotional function; *CF*, cognitive function; *SF*, social function; *P-Fat*, physical fatigue; *E-Fat*, emotional fatigue; *C-Fat*, cognitive fatigue; *Pain*, pain level (BPI); *Imp*, impairment through pain (BPI)

### Overlap of the profiles

The profiles from the first and the second LPA do not appear independent from each other. Instead, we see a relationship between PAHCO and symptom burden, meaning that patients assigned to the profile with low PAHCO appear to be more likely to be assigned to the profile of patients with higher symptom burden as well. Conversely, the low PAHCO profile constitutes exclusively from patients who were also assigned to symptom profiles two and three. Accordingly, those patients who were assigned to the profile with high PAHCO would more likely suffer less from symptoms. The high PAHCO profile constitutes to 58% of patients from symptom profile one which is the profile with the lowest symptom burden (see Online Resource 4).

### Analysis of variance (ANOVA)

The ANOVA yielded low *p*-values between PAHCO profiles for PA (*F*(2,87) = 3.1951, *p* = 0.046) and the number of comorbidities (*F*(2,94) = 3.197, *p* = 0.045), with those in P profile one displaying higher PA on average and lower comorbidities than the other two profiles. Concerning the symptom profiles, the number of treatment lines (*F*(2, 82) = 4.725, *p* = 0.011). In S-profile one, the number of treatment lines was lower compared to the other profiles. All other outcomes yielded large *p*-values (*p* > 0.10); however, there was a significant negative trend between symptom burden and leisure time PA (*p* = 0.0396).

## Discussion

To the best of our knowledge, this was the first study to investigate PAHCO levels and symptom burden with the intention to stratify MM patients with their different needs for exercise support. Our analysis suggests that based on the PAHCO and symptom burden, different groups of MM patients exist requiring individualized support, exercise recommendations, and different supervision modalities, given to their PAHCO and symptom profile.

A high PAHCO level indicates that a person has the necessary competences to implement a physically active lifestyle. Accordingly, MM patients in our analyses could be assigned to three distinct PAHCO groups. The first group (P-profile one, 46% of patients) represents patients with high PAHCO, the second group (P-profile two, 48%) represents patients with moderate PAHCO, and the third group (P-profile three, 5%) represents patients with low PAHCO. The ANOVA showed a statistically significant difference for number of comorbidities, with less comorbidities in the P-profile one patients with high PAHCO. Further, a statistically significant difference for PA levels with higher PA level for the P-profile one patients could be observed. We found no difference between profiles for number of treatment lines, age, and gender.

Stratifying patients according to their PAHCO level would help exercise therapists to provide appropriate supervision and educational instructions tailored particularly to every patient’s needs. Patients would presumably feel more motivated and empowered to engage in exercise and PA independently. For example, patients with moderate and/or low PAHCO (P-profiles two and three) could be offered supervised exercise programs in which an educational component is integrated, e.g., increasing knowledge about positive exercise effects, learning proper exercise techniques and forms of exercise, as well as raising motivation and volition (e.g., goal setting, action planning) [23, 42]. Especially for patients with low PAHCO, experienced-based learning could be very helpful to overcome concerns and uncertainties regarding exercise and PA due to therapy-related side effects such as polyneuropathy, pain, or high fracture risk [17]. For patients with high PAHCO (P-profile one), providing information about the positive effects of exercise and current exercise recommendations according to the ACSM guidelines [2] would probably be sufficient because they are already physically active and have less comorbidities that need to be considered when prescribing exercise. Hence, the application of the 5-A framework (ask, advise, agree, assist, arrange) might be useful to address ones needs corresponding with the PAHCO level. The 5-A framework is recommended for health behavior counseling [43]. Furthermore, home-based training after a supervised introduction could be an option for patients with high PAHCO and elevated fracture risk.

The necessity of increasing PAHCO in MM patients is supported by the literature. According to a survey in 289 MM patients, 52% reported to have insufficient control over physical side effects [44]. This finding corresponds with the low CC displayed in our analysis in P-profiles two and three, which incorporates 54% of the sample. Comparing the PAHCO levels to our cohort of 398 mixed cancer patients from the USA, our German MM patients have lower levels of MR (75 vs 67), a comparable level of CC (67 vs 67), and a higher level of SC (44 vs 67) [26].

Regarding the positive association of PAHCO and PA, our results are in accordance with our previous study in cancer patients, in which small to moderate positive associations between PAHCO dimensions and PA could be observed [26]. Further, the highest amount of PA was observed by individuals with high levels of self-regulation competence [21]. A recent study in multiple sclerosis patients could also show that self-regulation competence was a significant factor for a positive PA level and personal health [45].

Since not only adequate supervision and educational support may enhance the PA level but also the adaption to individual symptom profile is necessary when prescribing exercise, we also included a LPA of symptom burden in our analyses. Here, our analyses revealed three different groups of patients. Patients in the first group (S-profile one, 46%) display low symptom burden, including high physical functioning, low CRF, and low psychological strain, resulting in high QOL. Patients in the S-profile two (36%) group display medium symptom burden, including rather high CRF level and patients in the S-profile three (18%) show very high symptom burden, including high physical and psychological strain and CRF level, resulting in low QOL.

Regarding the symptom burden, patients in the S-profile three (very high symptom burden) show a high demand of supervised exercise tailored specifically to address the most distressing side effects. To attain the most effective exercise recommendation for a specific side effect, e.g., CRF, actual ACSM Guidelines should be followed [2] and the exercise program needs to be adapted to the actual symptoms. In contrast to this group, the S-profile one patients (very low symptom burden) only need little support and a standard/general exercise recommendation may be sufficient [2].

Looking at further potentially influencing variables, our ANOVA analysis revealed that the S-profile one patients with least symptom burden had significant less number of treatment lines than the other groups. The PA level, number of comorbidities, age, and gender seem to have no influence on symptom profiles.

Even though pain was not a large problem in our cohort, bone pain in MM is associated with bone disease [10] and 80–85% of newly diagnosed MM patients already suffer from bone disease [46]. Therefore, safety of exercise in MM patients with high fracture risk due to critical bone status has to be ensured. Even if a review demonstrates that exercise in cancer patients with stable bone metastases is safe [47], and guidelines for exercise with bone metastases exist [48], the situation for MM patients with multiple, frequently unstable bone lesions is more complex and has hardly been investigated thus far. Only one study could demonstrate the safety and feasibility of exercise in a group of cancer patients with unstable bone metastases [49]. However, first studies in MM patients have shown that exercise programs are feasible despite the high fracture risk, when highly individually adapted [13, 14]. Interestingly, a study in patients with bone metastases could show a positive association between exercise and bone pain during and after radiation therapy [50]. Actual recommendations for exercise with bone metastases include assessing the stability of the spine and long bones prior to entering any exercise program or a counseling session in order to provide safe exercise recommendations [48]. At the NCT in Heidelberg, we established a special clinic for MM patients, including an extensive evaluation of the actual fracture risk. Our multidisciplinary clinic comprises orthopedic, medical, and exercise expertise and tailored exercise advice is given, including 1:1 introduction of a selected exercise program. Furthermore, we offer a supervised or web-based exercise program to MM patients or/and we refer to an exercise specialist or physiotherapist close to the patient homes for supervised exercise via our network OncoActive.

In accordance with our results, a previous study on symptom burden in MM patients could show a direct association between high symptom levels, e.g., CRF, bone pain, and low levels of QOL; furthermore, the symptom severity level was a strong predictor of physical functioning. This study classified patients in asymptomatic, mild, moderate, and severe symptom levels. Compared to our MM patients, our S-profile three patients with the highest symptom level lay below the severely symptomatic patients of the other study. However, our S-profile one patients with low symptom burden display higher mean values than the asymptomatic group of that study [7].

If we consider the association between the PAHCO level and the symptom burden, our results indicate that there is a large proportion of patients with relatively low PAHCO level (P-profiles two and three) and high symptom burden (S-profile three), while 58% of the patients with high PAHCO display low symptom burden (S-profile one). This result indicates that most patients with high symptom burden not only need higher support due to their symptoms but also because of their low PAHCO. Studies show that MM patients want to be physically active and are interested in exercise programs but different barriers seem to prevent them from exercising. [17, 18, 20] Therefore, a precise screening for symptoms and PAHCO level is necessary to estimate the amount of care and educational support required for the individual patient.

Besides the positive effects PA and exercise have on side effects and QOL, promoting PA in MM patients seems of great clinical importance since a recent retrospective study showed that physically active MM patients have better clinical outcomes, including treatment tolerance and overall and progression-free survival [51]. Therefore, more personalized approaches increase the effectiveness of exercise counseling, which is necessary to improve the PA behavior of MM patients. Furthermore, the stratification process has also an economic aspect, may prevent misallocation of resources by providing especially patients in need with the highest level of support, and prevents an undersupply of these highly demanding patients.

Our analyses have several strength and limitations. For the LPA regarding PAHCO and symptom burden, all groups showed high assignment probabilities which means that the groups are very distinct from each other and there is a very small likelihood of false assignments of the individuals to the groups. However, when it comes to the PAHCO, P-profile three is borderline spurious according to established methodical recommendations, since it makes up only 5% of the cohort [39]. Nevertheless, we maintained the three-profile solution based on two considerations. First, we believe that our data underestimates the size of this group that would be expected in a more representative sample of MM patients, including also in-patients or more patients under intensive treatment. Most of the patients we approached were at the NCT Heidelberg for an ambulatory control visit and therefore not under intensive treatment, such as stem cell transplantation. The second argument is a more practical one. Given that the individuals in group three display very low levels of PAHCO, these patients would receive more attention and supervision pertaining to exercise. We see this risk of a potential oversupply to a few individuals as less problematic than the opposite scenario where patients with very low PAHCO would not receive sufficient care unless they were assigned to a profile with higher PAHCO.

The cross-sectional character of the study does not allow for causal conclusions; therefore, our inferences about the efficacy of educative interventions are speculative and need to be investigated in a confirmatory trial. Further research is necessary in order to investigate our proposed screening for PAHCO and symptom burden in an experimental approach. Furthermore, the PAHCO questionnaire is a generic instrument; however, a first validation study in cancer patients showed an excellent fit of the measurement model [26]. Moreover, one of the major sub-competences of the PAHCO model, the movement competence, was not assessed by our questionnaire. Here, in further research the newer versions of the PAHCO instruments should be used [52]. Lastly, our latent variables reflect only a subjective perception and no objective variable, e.g., physical performance was assessed.

## Conclusion

From an exercise oncology view, MM patients are a highly complex population with a wide range of therapy-related side effects, high fracture risk due to osteolytic bone lesions, and different PA levels. Therefore, implementing exercise programs or providing exercise recommendations is challenging. Consequently, it may be very effective to stratify patients in order to provide personalized exercise recommendations.

We observed three different levels of PAHCO that can be used to assess the level of support patients need to initiate and maintain an exercise program. After assessment of PAHCO level, a more personalized exercise counseling is possible by including educational instructions tailored particularly to every patient’s needs. Furthermore, our data points to the importance of considering different symptom clusters and selecting exercise content matching to the individual symptom load. Besides stratifying MM patients according to their PAHCO and symptom burden, it is also essential to assess actual fracture risk. Here, the actual guidelines for exercise with bone metastases should be followed [48]. Based on our findings, applying these three “screening” tools (PAHCO, symptom, and fracture risk) can support exercise oncology specialist to individually prepare an exercise training program or exercise counseling in MM patients.

### Supplementary Information

Below is the link to the electronic supplementary material.

Supplementary file1 (PDF 43 KB)

Supplementary file2 (PDF 109 KB)

Supplementary file3 (PDF 55 KB)

Supplementary file4 (PDF 95 KB)

Supplementary file5 (PDF 94 KB)

## Data Availability

The datasets generated during and/or analyzed during the current study are available from the corresponding author on a reasonable request.
