# Supplementary material for: Physical activity-related health competence and symptom burden for exercise prescription in patients with multiple myeloma: a latent profile analysis
Source: Ann Hematol. 2023 Jun 24;102(11):3091–102. doi: 10.1007/s00277-023-05326-y (PMC10567830; doi:10.1007/s00277-023-05326-y)
Supplement: Supplementary file 2 — Supplementary file2 (PDF 109 KB) [file 277_2023_5326_MOESM2_ESM.pdf]

Physical Activity-related Health Competence and Symptom Burden for Exercise Prescription in Patients with Multiple Myeloma: A Latent Profile Analysis

Kuehl, Rea[1]; Koeppel, Maximilian[1]; Goldschmidt, Hartmut[2], Maatouk, Imad[3,4], Rosenberger, Friederike[1,5], Wiskemann, Joachim[1]

[1]Working Group Exercise Oncology, Division Medical Oncology, National Center for Tumor Diseases (NCT) Heidelberg, Germany
[2]Department of Internal Medicine V, University Hospital Heidelberg and National Center for Tumor Diseases (NCT) Heidelberg, Germany
[3]Department of General Internal Medicine and Psychosomatics, University Hospital Heidelberg, Germany
[4]Section of Psychosomatic Medicine, Psychotherapy and Psychooncology, Department of Internal Medicine II, Julius-Maximilian University Wuerzburg, Wuerzburg, Germany
[5]Division of Health Sciences, German University of Applied Sciences for Prevention and Health Management, Saarbruecken, Germany

joachim.wiskemann@nct-heidelberg.de

Online Resource 2. Information Criteria for LPA Model Comparison

PAHCO-Profiles								
Profiles	AIC	AWE	BIC	CAIC	CLC	KIC	SABIC	ICL
1	2688,89	2747,78	2704,34	2710,34	2678,89	2697,89	2685,39	-2704,34
2	2625,66	2725,69[a]	2651,40	2661,40	2607,12	2638,66	2619,83	-2669,07
3	2587,19	2727,56[b]	2623,24[b]	2637,24[b]	2560,92	2604,19	2579,03	-2639,42[b]
4	2563,04[a]	2743,89	2609,38[a]	2627,38[a]	2528,87[b]	2584,04[a]	2552,54[a]	-2621,48[a]
5	2571,04[b]	2792,70	2627,68	2649,68	2528,67[a]	2596,04[b]	2558,22[b]	-2662,49

PAHCO: Physical Activity-Related Health Competence, AIC: Akaike Information Criterion, AWE: Approximate Weight of Evidence Criterion, BIC: Bayesian Information Criterion, CAIC: Consistent Aikake information Criterion, CLC: Classification Likelihood Criterion, ICL: Integrated completed likelihood, KIC: Kashyap Information Criterion, SABIC: Sample Size adjusted Bayesian Information Criterion
[a]Best Model according to fit index, [b]Second best model according to fit index

Symptom Burden-Profiles								
Profiles	AIC	AWE	BIC	CAIC	CLC	KIC	SABIC	ICL
1	9651,86	9946,42	9725,14	9755,14	9593,86	9684,86	9630,49	-9725,14
2	9227,18	9680,01[a]	9339,54	9385,54	9137,08	9276,18	9194,42	-9342,35
3	9073,32	9684,27[b]	9224,76[a]	9286,76[a]	8951,25	9138,32[b]	9029,16	-9227,13[a]
4	9064,00[b]	9833,18	9254,50	9332,52[b]	8909,87[b]	9145,00	9008,45[b]	-9260,96
5	9020,55[a]	9947,86	9250,16[b]	9344,16	8834,45[a]	9117,55[a]	8953,60[a]	-9255,03[b]

AIC: Akaike Information Criterion, AWE: Approximate Weight of Evidence Criterion, BIC: Bayesian Information Criterion, CAIC: Consistent Aikake information Criterion, CLC: Classification Likelihood Criterion, ICL: Integrated completed likelihood, KIC: Kashyap Information Criterion, SABIC: Sample Size adjusted Bayesian Information Criterion
[a]Best Model according to fit index, [b]Second best model according to fit index
