# Supplementary material for: Physical activity-related health competence and symptom burden for exercise prescription in patients with multiple myeloma: a latent profile analysis
Source: Ann Hematol. 2023 Jun 24;102(11):3091–102. doi: 10.1007/s00277-023-05326-y (PMC10567830; doi:10.1007/s00277-023-05326-y)
Supplement: Supplementary file 3 — Supplementary file3 (PDF 55 KB) [file 277_2023_5326_MOESM3_ESM.pdf]

Physical Activity-related Health Competence and Symptom Burden for Exercise Prescription in Patients with Multiple Myeloma: A Latent Profile Analysis

Kuehl, Rea[1]; Koeppel, Maximilian[1]; Goldschmidt, Hartmut[2], Maatouk, Imad[3,4], Rosenberger, Friederike[1,5], Wiskemann, Joachim[1]

[1]Working Group Exercise Oncology, Division Medical Oncology, National Center for Tumor Diseases (NCT) Heidelberg, Germany
[2]Department of Internal Medicine V, University Hospital Heidelberg and National Center for Tumor Dis-eases (NCT) Heidelberg, Germany
[3]Department of General Internal Medicine and Psychosomatics, University Hospital Heidelberg, Germany
[4]Section of Psychosomatic Medicine, Psychotherapy and Psychooncology, Department of Internal Medicine II, Julius-Maximilian University Wuerzburg, Wuerzburg, Germany[5]Division of Health Sciences, German University of Applied Sciences for Prevention and Health Management, Saarbruecken, Germany

joachim.wiskemann@nct-heidelberg.de

Online Resource 3

Tab.S3.1. Mean Assignment Probability for PAHCO-Profiles

		Patients assigned to Profile:		
		**High PAHCO**	**Moderate PAHCO**	**Low PAHCO**
**Average probability to belong to Profile**	**High PAHCO**	91 %	5 %	0 %
	**Moderate PAHCO**	9 %	95 %	2 %
	**Low PAHCO**	0 %	0 %	98 %

Tab.S3.2. Mean Assignment Probability for Symptom-Profiles

		Patients assigned to Profile:		
		**Low Burden**	**Moderate Burden**	**High Burden**
**Average probability to belong to Profile**	**Low Burden**	98 %	0 %	1 %
	**Moderate Burden**	0 %	100 %	0 %
	**High Burden**	2 %	0 %	99 %

Interpretation: In contrast to conventional cluster analysis where a person is deterministically assigned to a particular class, in latent profile analysis (LPA) a person is assigned to the profile which shows the highest assignment probability for this very person. Thus, the characteristics of a person could also fit to another profile displayed by an assignment probability larger than zero. The following tables describe the average assignment probabilities per Profile. The diagonal describes the average assignment probability used to assign a person to the profile they were eventually assigned to, the off-diagonals describe how well the average person of a the profile named in the column would fit to the other profiles. E.g. in Table S3.1 The average assignment probability of the individuals in Profile 1 is 91%. While their average assignment probability to Profile 2 would be 9%.
Tab.S3.1. Mean Assignment Probability for PAHCO-Profiles
