# Supplementary material for: Physical activity-related health competence and symptom burden for exercise prescription in patients with multiple myeloma: a latent profile analysis
Source: Ann Hematol. 2023 Jun 24;102(11):3091–102. doi: 10.1007/s00277-023-05326-y (PMC10567830; doi:10.1007/s00277-023-05326-y)
Supplement: Supplementary file 4 — Supplementary file4 (PDF 95 KB) [file 277_2023_5326_MOESM4_ESM.pdf]

Physical Activity-related Health Competence and Symptom Burden for Exercise Prescription in Patients with Multiple Myeloma: A Latent Profile Analysis

Kuehl, Rea[1]; Koeppel, Maximilian[1]; Goldschmidt, Hartmut[2], Maatouk, Imad[3,4], Rosenberger, Friederike[1,5], Wiskemann, Joachim[1]

[1]Working Group Exercise Oncology, Division Medical Oncology, National Center for Tumor Diseases (NCT) Heidelberg, Germany
[2]Department of Internal Medicine V, University Hospital Heidelberg and National Center for Tumor Dis-eases (NCT) Heidelberg, Germany
[3]Department of General Internal Medicine and Psychosomatics, University Hospital Heidelberg, Germany
[4]Section of Psychosomatic Medicine, Psychotherapy and Psychooncology, Department of Internal Medicine II, Julius-Maximilian University Wuerzburg, Wuerzburg, Germany
[5]Division of Health Sciences, German University of Applied Sciences for Prevention and Health Management, Saarbruecken, Germany

joachim.wiskemann@nct-heidelberg.de

Online resource 4. Composition of PAHCO Classes in regards to symptom classes in % (~~N~~n)

		Symptom Burden		
		Profile 1	Profile 2	Profile 3
PAHCO	Profile 1	58 (21)	33 (12)	8 (3)
	Profile 2	23 (10)	55 (24)	23 (10)
	Profile 3	0 (0)	50 (2)	50 (2)

PAHCO: Physical activity-related health competences,
